# Differences in gut microbiota composition in finishing Landrace pigs with low and high feed conversion ratios

**DOI:** 10.1007/s10482-018-1057-1

**Published:** 2018-03-01

**Authors:** Zhen Tan, Yuan Wang, Ting Yang, Hong Ao, Shaokang Chen, Kai Xing, Fengxia Zhang, Xitong Zhao, Jianfeng Liu, Chuduan Wang

**Affiliations:** 1 National Engineering Laboratory for Animal Breeding, Key Laboratory of Animal Genetics, Breeding and Reproduction, Ministry of Agriculture, College of Animal Science and Technology, China Agricultural University, Beijing, China; 2 Institute of Animal Sciences, Chinese Academy of Agricultural Sciences, Beijing, China; 3 Beijing General Station of Animal Husbandry, Beijing, China

**Keywords:** Feed conversion ratio (FCR), Gut microbiota, Microbial community, Pigs

## Abstract

The goal of this study was to evaluate the microbial communities in the gut and feces from female finishing Landrace pigs with high and low feed conversion ratio (FCR) by 16S rRNA gene amplicon sequencing. Many potential biomarkers can distinguish between high and low FCR groups in the duodenum, ileum, cecum, colon, and rectum, according to linear discriminant analysis effect sizes. The relative abundance of microbes were tested by Mann–Whitney test between the high and low FCR groups in different organs: *Campylobacter*, *Prevotella* and *Sphaerochaeta* were different in the duodenum (*P* < 0.05); *Sanguibacter*, *Kingella* and *Anaeroplasma* in jejunum; *Anaeroplasma, Arthrobacter, Kingella, Megasphaera* and SMB53 in the ileum; *Butyricicoccus, Campylobacter, Mitsuokella,* and *Coprobacillus* in the cecum; *Lactococcus* and *Peptococcus* in the colon; *Staphylococcus* in the rectum; and *Rothia* in feces. The prevalence of microbial genera in certain locations could potentially be used as biomarkers to distinguish between high and low FCR. Functional prediction clustering analysis suggested that bacteria in the hindgut mainly participated in carbohydrate metabolism and amino acid metabolism, and different in the relative abundance of metabolic pathways, as predicted from the microbial taxa present, were identified by comparing the high and low groups of each location. The results may provide insights for the alteration of the intestinal microbial communities to improve the growth rate of pigs.

**Electronic supplementary material:**

The online version of this article (10.1007/s10482-018-1057-1) contains supplementary material, which is available to authorized users.

## Introduction

Because of the rapid development of metagenomic studies, the gut microbiota from a variety of animals have been studied extensively in recent years for their role in disease causation and gut health maintenance, which has a marked influence on the health and performance of domestic livestocks. Shaped by genetic and environmental factors, especially diet, gut microbial diversity in pigs is also affected by the breed (Yang et al. 2014), the animal’s growth stage (Kim et al. 2015), and the intestinal segment (Kim and Isaacson 2015) of pigs.

Gut microbes share an essential and symbiotic relationship with their host. Microbes assist the host in maximizing the nutritional value of its diet. The large intestinal tract harbors more species and greater quantities of microorganisms than the small intestine, and the predominant species are different. One study reported that *Anaerobacter* and *Turicibacter* were the dominant genera in the ileum of 3-month-old pigs, while *Prevotella*, *Oscillibacter*, and *Succinivibrio* were prevalent in the colon (Looft et al. 2014). The large intestine plays the dominant role in microbial fermentation, where the resident microbiota decompose polysaccharides (such as resistant starch and dietary fiber) that are poorly hydrolyzed by enzymes in the small intestine (Louis et al. 2007; Schwiertz et al. 2010). The main products of the microbial fermentation of carbohydrates under anaerobic conditions are short-chain fatty acids (SCFAs), such as acetate, propionate, and butyrate (Macfarlane and Gibson 1997), which can be utilized by the host. In the epithelial cells of the colon, ketone bodies and carbon dioxide are produced by the metabolism of butyrate (Louis et al. 2007). The SCFAs produced by bacteria provide an additional source of energy for the body.

The gut microbiota has been shown to be involved in regulating the energy-harvesting efficiency and improving the energy-harvesting capacity of the host (Turnbaugh et al. 2006). These factors are associated with body weight gain (Kim et al. 2015; Kim and Isaacson 2015; Looft et al. 2012, 2014). Along with genetic changes, gut physiology and intestinal flora can affect feed efficiency (FE) (Lumpkins et al. 2010). Increased knowledge of the community structure and functional capacity of the gut microbiota helps to reveal relationships between microbial functions and the host’s physiology and metabolism.

Revealing the taxonomic composition and functional capacity of the gut microbiota and their interaction with the host should facilitate understanding of the roles they play in the host, and may improve pork production by identifying the component of FE associated with microorganisms. A study of the fecal microbiome in pigs of different fat content found that the cecal microbiome has the strongest ability to degrade xylan, pectin, and cellulose (Yang et al. 2016). Furthermore, taxonomy and functional capacity of fecal microbiota were determined in low and high feed conversion ratio (FCR) broilers (Singh et al. 2014).

Feed accounts for more than 60% of the costs of pig husbandry. Therefore, improving FE is one of the major ways to reduce costs in the pig farming industry. FE can be measured by the FCR. The FCR is the total weight of feed intake divided by the weight gained during a specified period. Thus, an animal with a high FCR is less efficient at converting feed into body mass than one with a low FCR. Previous studies have indicated that the heritability (the ratio of the genetic variance to the phenotype variance) of FCR is 0.13–0.31 (Gilbert et al. 2012; Jing et al. 2015). In addition, variation in feed-conversion efficiency is closely related to the genetic diversity of the gut microbiota (Singh et al. 2012, 2014; Yan et al. 2017). Therefore, the diversity of the gut microbiota is a factor in animal productivity, even under the same rearing conditions. In previous studies, we found that some probiotics, such as *Lactobacillus,* tend to be enriched in the cecal microbes and colonic microbes that provide high FE, compared to lower FE animals that had a higher proportion of *Prevotella* (Tan et al. 2017a, b). Functional analysis revealed that differentially expressed genes affect the host’s energy absorption mainly through pyruvate-related metabolism in cecal microbiota. Pathways mediating metabolism of cofactors and vitamins enriched in colonic microbiota of low FE animals might be linked to the consumption of carbohydrates that were incompletely digested before reaching the colon.

The composition of gut bacteria with difference FE between individuals related to metabolic changes, and ultimately to swine health and performance, is still unclear. We investigated the microbial communities in the gut contents and feces from female finishing Landrace pigs with high and low FCR using 16S rRNA gene amplicon sequencing. The abundance of the different bacterial populations comprising the microbiota were compared to determine the differences between gut locations (duodenum, ileum, jejunum, cecum, colon, rectum, feces) of the high and low FCR groups. We then determined whether the presence of certain bacteria is correlated with pig production performance.

## Materials and methods

### Animal experiments and DNA extraction

120 female Landrace pigs were housed in an environmentally controlled room (ten pigs in each pen), and given the same corn-soybean commodity diet without antibiotics or medicines. Clean water was provided ad libitum throughout the experiment. FCR was determined from feed intake and body weight, which was recorded from 120 to 165 days of age using a Velos (Nedap co., LTD, Groenlo, Netherland) automated individual feeding system that recognized an electronic ear mark. Individuals were ranked by FCR, and there was a significant difference between the high and low end (Supplementary Fig. 1). We defined the L group as individuals with low FE and high FCR values, and the H group as individuals with high FE and low FCR values (20 animals each). Two full-sibling pairs and two half-sibling pairs were selected, such that the siblings within each pair had opposite FCR phenotypes (Supplementary Table 1).

Fresh fecal materials were collected from each individual on day 165 and kept frozen in liquid nitrogen. The chosen pigs were euthanized on day 166, and digesta samples were collected from the duodenum, jejunum (middle section), ileum (distal part), cecum, colon (middle section), and rectum (distal part) within 30 min of euthanizing. All methods were in accordance with the guidelines approved by the Quality Supervision, Inspection, and Quarantine of the People’s Republic of China (GB/T 17236–2008). The Animal Welfare Committee of China Agricultural University approved all experimental protocols (permit number: DK996).

All samples were collected in sterile tubes, and then stored in liquid nitrogen until analysis. DNA was extracted and purified using a QIAamp DNA Stool Mini Kit (Qiagen Ltd., Germany) following the manufacturer’s instructions. Adequate quantities of high-quality genomic DNA were extracted, and the concentration of DNA was measured using a UV–Vis spectrophotometer (NanoDrop 2000c, USA).

### 16S rRNA gene sequencing

The V3–V4 region of the 16S rRNA gene was amplified (341F–806R) by polymerase chain reaction (PCR) (Kozich et al. 2013) with universal bacterial 16S rRNA gene PCR amplicon primers. All PCR reactions were carried out in 30 μL reaction volumes with 15 μL of Phusion^®^ High-Fidelity PCR Master Mix (New England Biolabs). Mixed PCR products were purified using a GeneJET Gel Extraction Kit (Thermo Scientific) following the manufacturer’s instructions. Sequencing libraries were generated using an NEB Next^®^ Ultra™ DNA Library Prep Kit for Illumina (NEB, USA) following the manufacturer’s recommendations. The library was sequenced on an Illumina MiSeq platform, and 250 bp paired-end reads were generated.

### Data analysis

#### Paired-end read assemblies and quality control

Paired-end reads from the original DNA fragments were merged using FLASH (Lozupone et al. 2011). Paired-end reads (tags) were assigned to each sample according to the unique barcodes. Raw tags were quality controlled by QIIME (Caporaso et al. 2010). Low quality (Phred score < 20) base sites were truncated when the continuous low-quality base number reached three. Tags were filtered out of which contents continuous high-quality base lengths no more than three quarters of the whole tags. Chimeric sequences were removed by UCHIME.

#### Operational taxonomic unit (OTU) clusters and species annotation

Sequences analyses were performed using the QIIME pipeline (version 1.8.0) (Caporaso et al. 2010). Sequences with ≥ 97% similarity were assigned to the same operational taxonomic units (OTUs), picked by UPARSE. OTUs were annotated with taxonomic information using the Ribosomal Database Project classifier (Edgar 2013). The relative abundance of taxa was determined according to the annotated taxonomic information. Microbiota in duodenum of H group were assigned as Hduodenum, Microbiota in duodenum of the L group were assigned as Lduodenum, etc.

#### Community distribution and functional annotation

A histogram was used to graphical represent the relative abundance of taxonomic groups from phylum to species. QIIME calculated both weighted and unweighted UniFrac distances, which are phylogenetic measures of beta diversity (Lozupone et al. 2011). A heatmap was plotted to cluster samples from the different groups. Phylum and family relative abundance were represented by stacked bar charts. We used unweighted UniFrac distances for principal coordinate analysis (PCoA) (Avershina et al. 2013). Linear discriminant analysis (LDA) effect size (LEfSe) was used for the quantitative analysis of biomarkers within different groups (Segata et al. 2011). All of the OTU functions were predicted by the Kyoto Encyclopedia of Genes and Genomes (KEGG) database, based on the structure of the gastrointestinal microbiota established using PICRUSt (Langille et al. 2013).

### Statistical analysis

To confirm differences in the abundances of individual taxonomies between the two groups, we carried out statistical analyses using R (http://www.R-project.org). Non-parametric tests (Mann–Whitney) were carried out to identify differences in microbial communities between the two groups. The data were deposited in the National Center for Biotechnology Information’s Short Read Archive under Accession No. SRR5038273.

## Results

### Bacterial diversity and composition of high and low FCR groups

After quality control and demultiplexing, more than 30,000 valid sequences were generated from each group (n = 4). The number of OTUs with a 97% identity cut-off was determined for each group. The average number of sequences in each group ranged from 31,463 to 37,355, with the number of OTUs from 1214 to 2352. Bacterial diversities were compared among each location (intestines and feces) using diversity and richness estimators (Table 1). Considering the number of OTUs, Ljejunum was higher than Hjejunum, Lileum exceeded Hileum, and Hcolon surpassed Lcolon. Except for the Simpson index, Lileum was more significant than Hileum in other α-index. Hcolon was higher than Lcolon in chao1 and ACE indices.

**Table 1:** Sequences, operational taxonomic unit (OTUs), and alpha diversity for each intestinal and fecal location in high and low food conversion ratio (FCR) groups

	Seqs	OTUs	Observed species	Goods coverage	ACE	Chao1	PD whole tree	Simpson	Shannon
Hduodenum	31916	1798	1854	0.932	3125	2972	145	0.965	7.852
Lduodenum	35356	1938	1901	0.945	3208	3024	153	0.924	7.06
*P* value	0.248	0.564	0.773	0.386	0.564	0.386	0.773	0.386	0.386
Hjejunum	31463	1580	1699	0.946	2925	2757	136	0.948	7.254
Ljejunum	37355	1742	1914	0.95	3418	3257	146	0.901	6.515
*P* value	0.564	0.043	0.043	0.248	0.149	0.149	0.083	0.083	0.149
Hileum	35968	1214	1285	0.961	2646	2441	111	0.926	5.692
Lileum	36264	2076	2228	0.942	3834	3649	162	0.963	7.637
*P* value	0.827	0.0495	0.0495	0.0495	0.0495	0.0495	0.0495	0.127	0.0495
Hcecum	33006	2144	2269	0.93	3902	3695	164	0.985	8.672
Lcecum	35427	2278	2311	0.939	3680	3546	162	0.99	8.939
*P* value	0.564	0.386	0.564	0.248	0.386	0.564	0.773	0.386	0.564
Hcolon	32762	2081	2315	0.928	3902	3783	165	0.99	8.909
Lcolon	32263	1986	1995	0.938	3199	3063	147	0.991	8.794
*P* value	0.773	0.386	0.021	0.248	0.021	0.021	0.021	0.564	0.773
Hrectum	36195	1923	2108	0.946	3350	3222	157	0.979	8.37
Lrectum	34675	2155	2293	0.934	3718	3570	163	0.993	9.057
*P* value	0.773	0.248	0.083	0.248	0.083	0.083	0.386	0.564	0.149
Hfeces	32532	2204	2167	0.937	3478	3293	155	0.987	8.832
Lfeces	33556	2352	2314	0.936	3543	3370	164	0.994	9.254
*P* value	0.386	0.564	0.564	0.773	1	0.564	0.248	0.149	0.083

n = 4 in each measurement; the observed species index shows the number of OTUs actually observed; ACE and Chao1 indices were used to estimate the number of OTUs and microbial richness; the goods coverage index was used to reflect the species coverage; the PD whole tree index was based on the phylogenetic tree; Shannon and Simpson indices were used to assess biodiversity

### Taxonomic composition

**Fig. 1:**
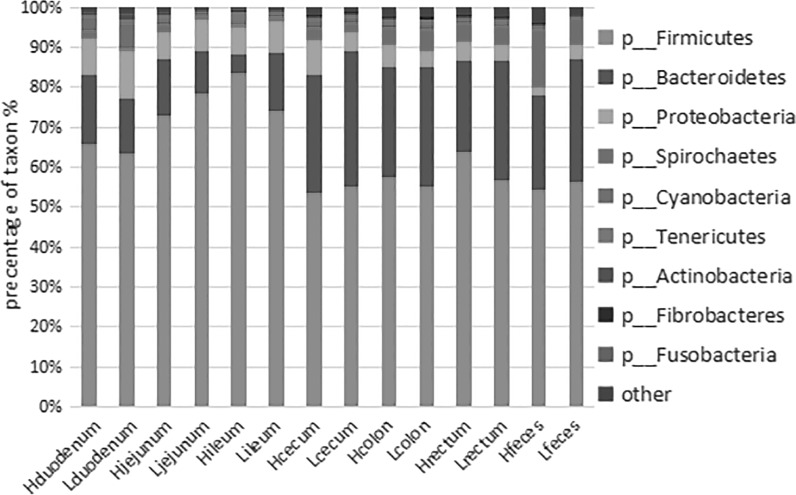
Average relative abundances of predominant bacteria at the phylum level in the intestinal digesta in high and low food conversion ratio (FCR) groups at each location

Firmicutes, Bacteroidetes, Proteobacteria, Spirochaetes, and Cyanobacteria were the top five phyla, regardless of gut location, and more than 95% of the sequences could be assigned to them (Fig. 1). The most abundant sequences detected at the phylum level were from the Firmicutes, comprising more than 50% of all the normalized reads. There was also taxonomic variation in microbial composition among the high and low FCR groups in every location.

**Fig. 2:**
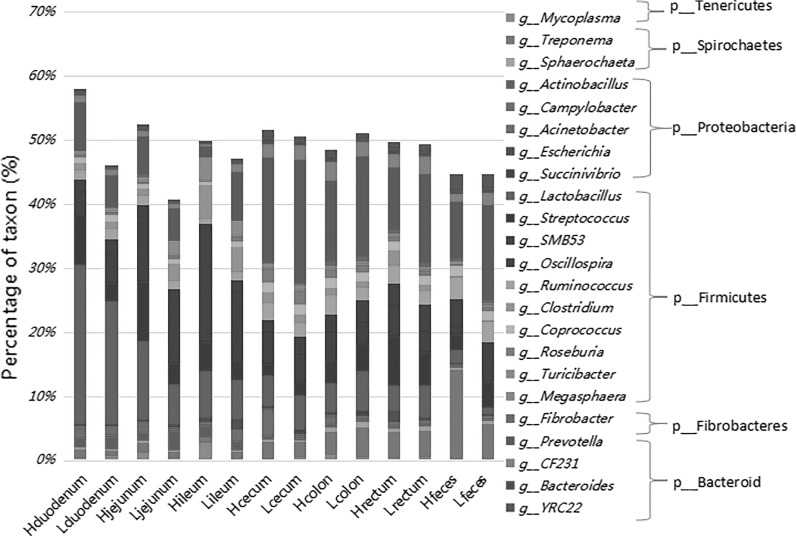
Average relative abundances of predominant bacteria at the genus level of the intestinal digesta in high and low food conversion ratio (FCR) groups at each location

At the genus level, unclassified bacteria in all locations accounted for 41.95–59.28% of the total reads (Fig. 2). *Lactobacillus* was the most abundant genus in Hduodenum, Lduodenum, and Hjejunum (24.81, 19.25, and 12.32%, respectively); the candidate genus SMB53 was the most abundant in Ljejunum, Hileum, and Lileum. *Prevotella* was the predominant genus in both the high and low FCR groups in the cecum, colon, rectum, and Lfeces locations.

**Fig. 3:**
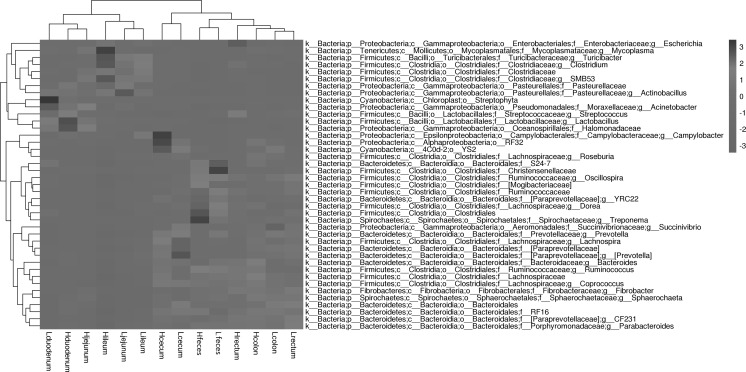
Heatmap hierarchical cluster analysis based on differentially abundant bacteria (operational taxonomic units (OTUs) at 97% identity) in high and low food conversion ratio (FCR) groups at different intestinal locations. The relative levels of abundance are depicted visually from red to blue; red represents the lowest abundance (min = − 3), whereas blue (max = 3) represents the highest level of abundance. (Color figure online)

We plotted a heatmap (Fig. 3) to determine microbial community similarities in the locations between the two groups. The heatmap shows that there are obvious differences between the anterior intestine (duodenum, jejunum, ileum) and the posterior segments (cecum, colon, rectum, feces).

**Fig. 4:**
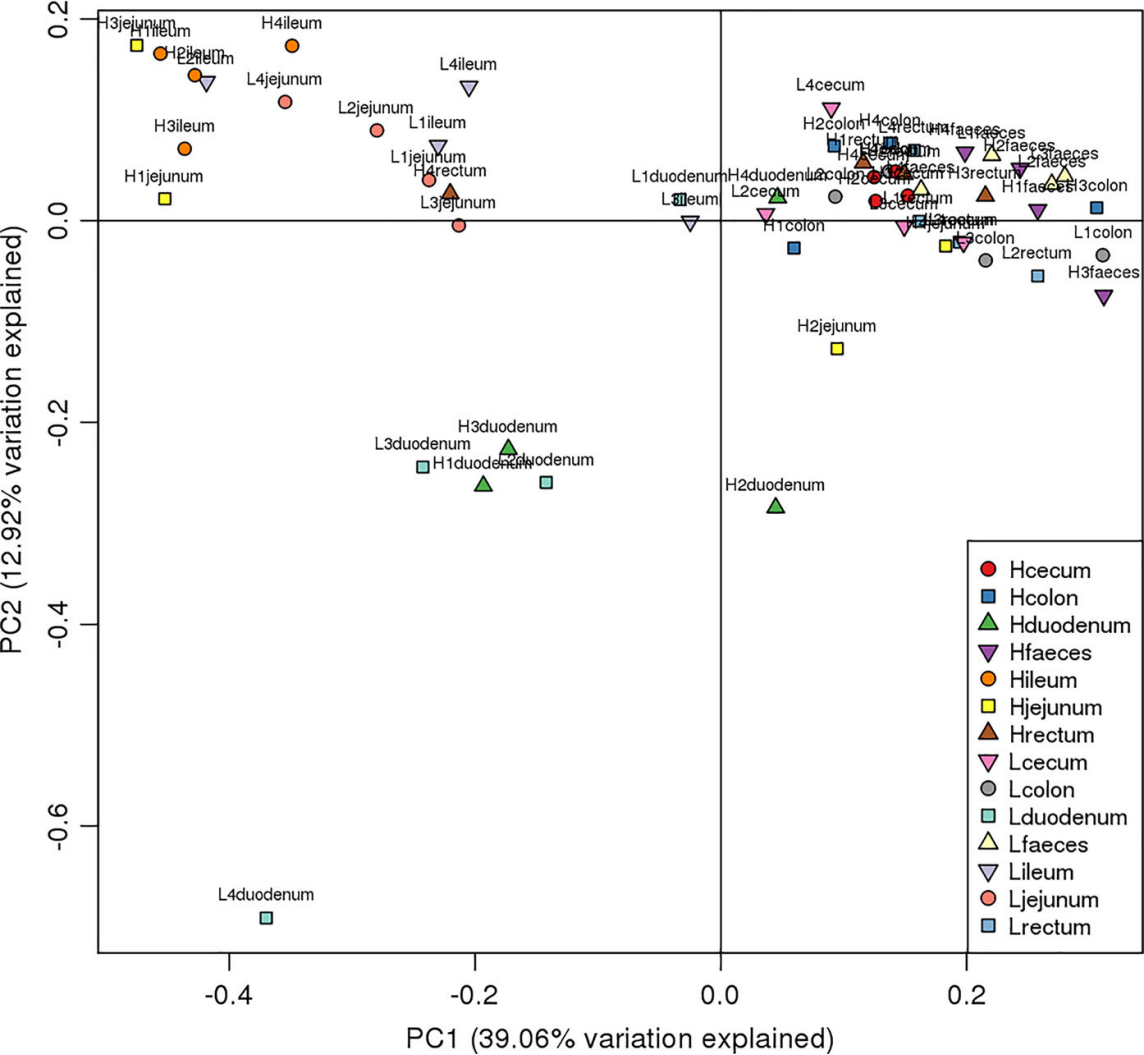
Principal coordinate analysis (PCoA) based on the weighted UniFrac distance of 16s rRNA of intestinal bacteria in the high and low food conversion ratio (FCR) groups

We used PCoA (Fig. 4) to compare the membership and structure of the samples at the genus level. The posterior segment samples had a more centralized distribution, and maintained a certain distance from anterior intestinal samples. However the L4duodenum sample was far away from the other three samples in the Lduodenum group, suggesting intra-group variation.

### Characterization of gut microbes in locations of the high and low FCR groups

In the anterior intestine (duodenum, jejunum, ileum), Firmicutes, Bacteroidetes, and Proteobacteria, were the three most abundant phyla. They constituted more than 90% of all phyla detected. Firmicutes accounted for over 60% in every group.

**Fig. 5:**
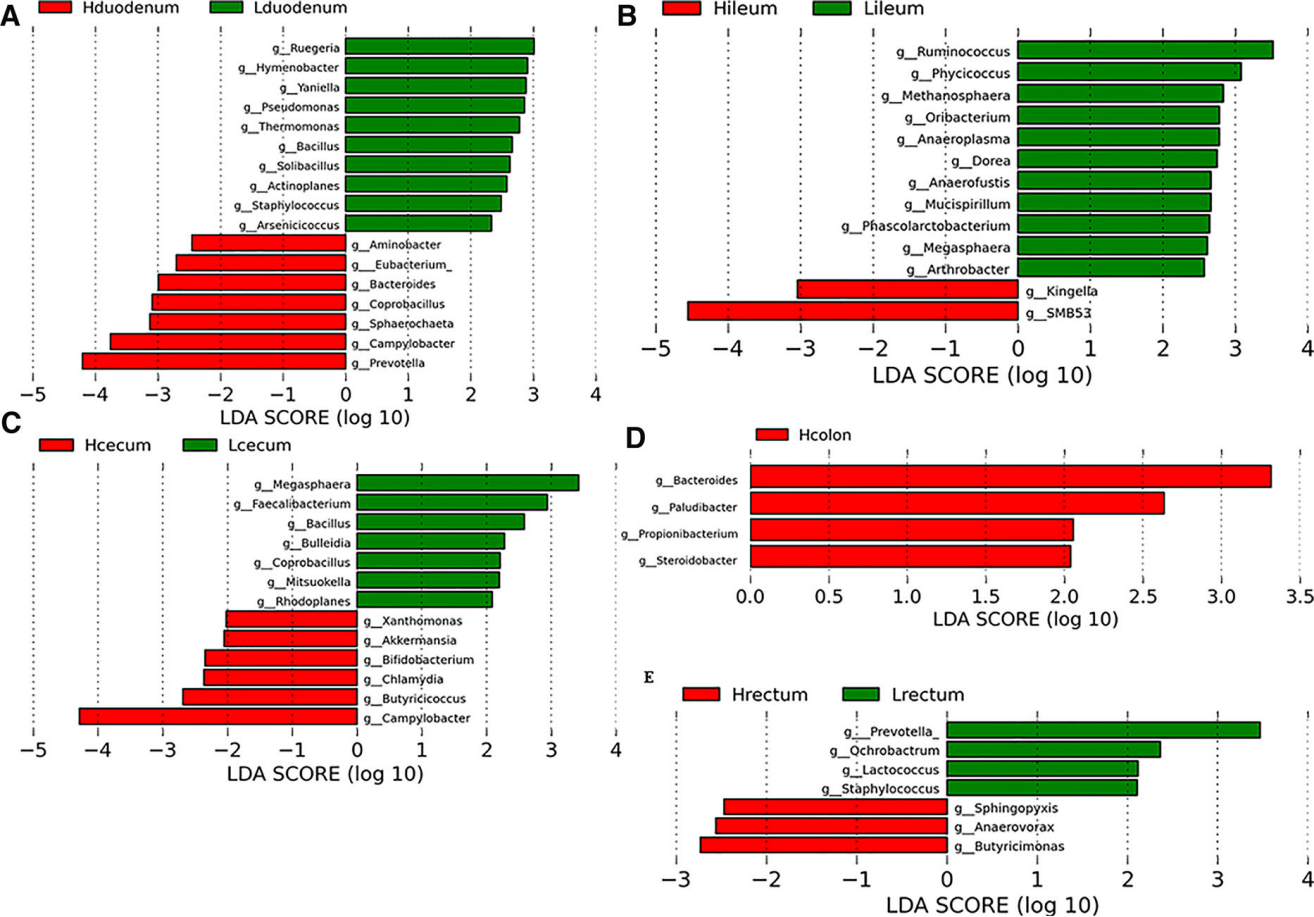
Linear discriminant analysis (LDA) effect size (LEfSe) results for microbiota of intestinal locations at the genus level. Histogram of the LDA scores computed for features differentially abundant in the duodenum (**a**), ileum (**b**), cecum (**c**), colon (**d**), and rectum (**e**) at the genus level among high and low groups (only genera LDA scores above 2 are shown)

At the genus level, the five dominant genera detected in both groups were: *Lactobacillus*, *Streptococcus*, *Prevotella*, SMB53, and *Oscillospira* (Fig. 2). Hduodenum had a higher relative abundance of the genera *Lactobacillus*, *Streptococcus*, and *Prevotella*; the relative ratios (Hduodenum/Lduodenum) were 1.55, 2.34, and 1.78, respectively. There was a higher abundance of *Campylobacter* and *Sphaerochaeta* in the Hduodenum microbiota (*P* < 0.05) than in the Lduodenum (Table 2). 14 genera were found to be potential biomarkers for distinguishing between high and low FCR groups; 8 genera were unique to Lduodenum and 6 were unique to Hduodenum (Fig. 5a).

**Table 2:** Bacterial genera with significantly different representations between the two food conversion ratio (FCR) groups (*P* < 0.05)

Location	Genus	Hgroup	Lgroup	*P* value
Duodenum	*Campylobacter*	0.012114	0.002359	0.028571
	*Prevotella*	0.067695	0.038038	0.028571
	*Sphaerochaeta*	0.003405	0.000668	0.028571
Jejunum	*Sanguibacter*	0.000066	0.000013	0.029401
Ileum	*Kingella*	0.000250	0.000000	0.021071
	*Anaeroplasma*	0.000484	0.001202	0.028571
	*Arthrobacter*	0.000042	0.000331	0.028571
	*Megasphaera*	0.000114	0.000908	0.028571
	*SMB53*	0.177427	0.108163	0.028571
Cecum	*Rhodoplanes*	0.000000	0.000069	0.021071
	*Megasphaera*	0.000461	0.004553	0.028571
	*Campylobacter*	0.040242	0.003385	0.028571
	*Butyricicoccus*	0.002668	0.001584	0.028571
	*Mitsuokella*	0.000067	0.000294	0.028571
Colon	*Coprobacillus*	0.000113	0.000038	0.028571
	*Lactococcus*	0.000069	0.000014	0.029401
	*Peptococcus*	0.000061	0.000130	0.028571
Rectum	*Staphylococcus*	0.000029	0.000105	0.028571
Feces	*Rothia*	0.000000	0.000072	0.021071

The significance (*P* value) was obtained by Mann–Whitney test

In the jejunum, the prevalent genera were *Lactobacillus*, SMB53, *Streptococcus*, and *Prevotella*. The genus SMB53 had a similar prevalence in both groups, but *Lactobacillus* was nearly twice as abundant in the high FCR group than in the low FCR group, and *Streptococcus* was almost three times more abundant (high 9.23%, low 3.19%). *Sanguibacter* was the only significantly different genus between the two groups (Table 2).

In the ileum, the top five most abundant genera were SMB53, *Lactobacillus*, *Clostridium*, *Prevotella*, and *Streptococcus*. The abundance of the genus *Lactobacillus* was similar in both groups, but the relative abundance ratios of *Prevotella* and *Oscillospira* (Hileum/Lileum) were 0.23 and 0.53, respectively. The abundance of *Kingella* and SMB53 was higher in Hileum than Lileum (*P* < 0.05). *Anaeroplasma*, *Arthrobacter*, and *Megasphaera* were less abundant in the Hileum group (*P* < 0.05) (Table 2). 11 genera were found to be potential biomarkers to distinguish high and low FCR groups by LEfSe analysis; 9 genera were more abundant in Lileum and 2 genera were more abundant in Hileum (Fig. 5B).

In the posterior intestine (cecum, colon, rectum, and feces), the four most abundant phyla, (Firmicutes, Bacteroidetes, Proteobacteria, and Spirochaetes) constituted more than 95% of all the phyla detected. Firmicutes accounted for over 50% in every group and Bacteroidetes accounted for more than 20%. *Prevotella* was the prominent genus in both high and low FCR groups in the lower intestines. The PCoA plot showed that the lower intestine samples had a central distribution, suggesting that they were similar to each other (Fig. 3).

In the cecum, 13 genera were potential biomarkers for distinguishing between high and low FCR groups by LEfSe analysis; there were 7 genera unique to Lcecum and 6 genera unique to Hcecum (Fig. 5c).

*Campylobacter* and *Butyricicoccus* were more abundant in the cecum microbiota of the high FCR group *(P* < 0.05) compared to the low FCR group. *Megasphaera, Mitsuokella*, and *Rhodoplanes* were more abundant in the cecum microbiota of the lower FCR (*P* < 0.05) (Table 2).

There was a higher prevalence of the genera *Coprobacillus* and *Lactococcus,* and a lower abundance of *Peptococcus* in the Hcolon microbiota (*P* < 0.05) than in the Lcolon microbiota (Table 2). Only 2 genera were potential biomarkers, and they were both more abundant in Hcolon: *Bacteroides* and *Paludibacter* (Fig. 5d).

The Mann–Whitney test on the rectum and feces microbiota revealed that Lrectum had a greater abundance of *Staphylococcus* and Lfeces had a greater abundance of *Rothia* between the high and low FCR groups. The PCoA plot showed that the samples from the rectum and feces were very similar. We identified 9 genera as potential biomarkers for distinguishing between Hrectum and Lrectum (Fig. 5e).

### Metabolic pathways of gut microbes in the high and low FCR group locations

**Fig. 6:**
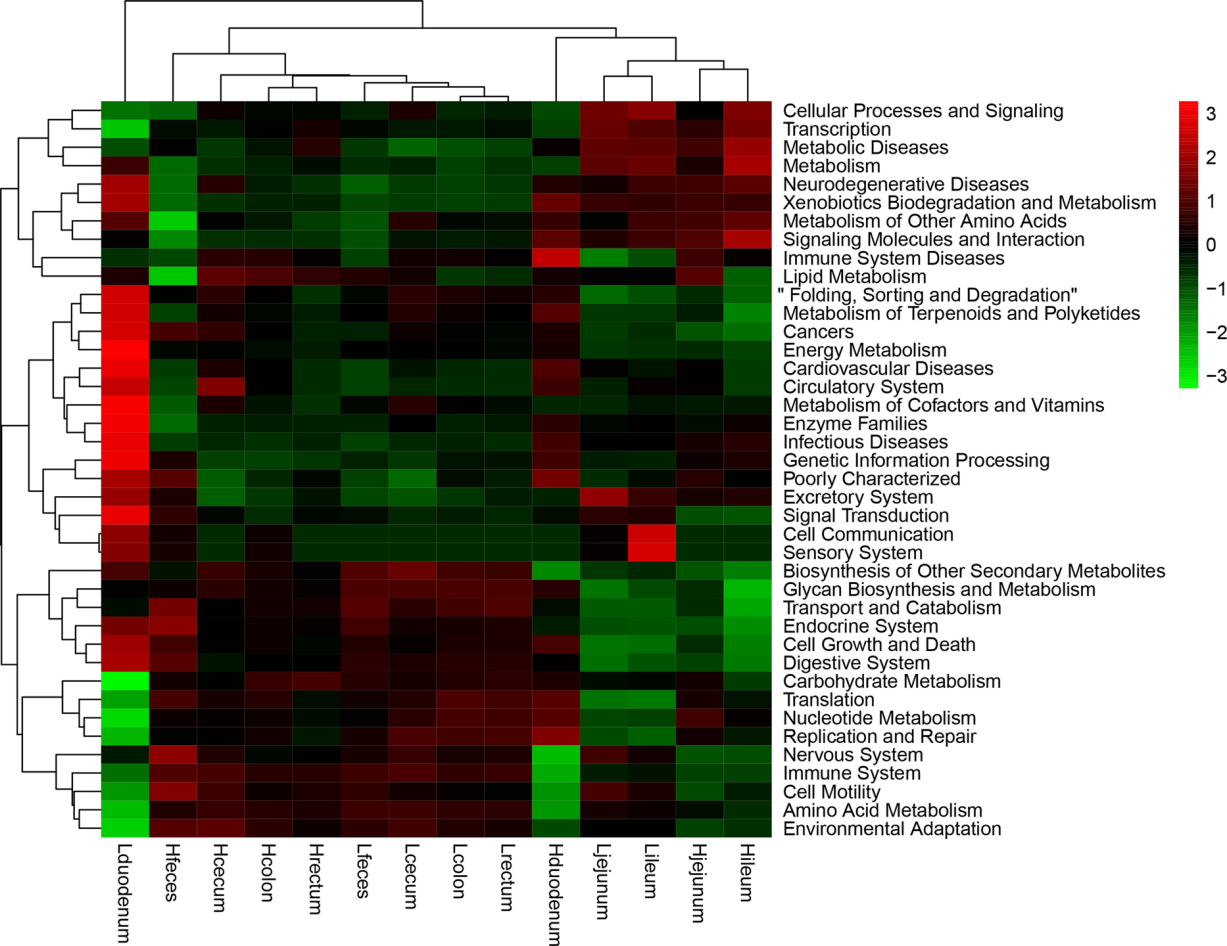
Comparison of KEGG pathways predicted by PICRUSt in high and low food conversion ratio (FCR) groups at different intestinal locations. The relative levels of abundance are depicted visually from red to blue; red represents the lowest abundance (min = − 3), whereas blue (max = 3) represents the highest level of abundance. (Color figure online)

A total of 300 third-level pathways, which belonged to 40 s-level classifications from the Kyoto Encyclopedia of Genes and Genomes (KEGG), were verified based on the structure of the gastrointestinal microbiota established, accroding to PICRUSt (Fig. 6). Other than the duodenal position, the clustering in the heatmap showed that the anterior and posterior segment of the intestine were in two functional categories, while the high and low groups had significant differences in the same location. The statistically significant third-level KEGG pathways were identified by LEfSe for each comparison. Except for duodenum, we found KEGG pathway classifications significantly differed between each comparison group (Supplementary Fig. 2).

## Discussion

Diet is the main factor that determines intestinal microflora in animals. Several other factors also influence animal performance (Kim and Isaacson 2015; Looft et al. 2014; Singh et al. 2014), and genetic factors cannot be ignored. The gut microbiota can vary significantly even in well-controlled environments, and many other factors could partly explain the observed differences (Nicholson et al. 2012; Parks et al. 2013; Turnbaugh et al. 2006; Yang et al. 2014). Close kinship pairs were chosen with two divergent phenotypes to reduce differences in genetic background. We defined the low group as individuals with low FE and high FCR values, and pigs with high FE and low FCR values were in the high group.

In this study, we characterized the microbiota of the gastrointestinal tract in relation to the FE of pigs during the finishing period, with the aim of determining microbial differences between the high and low FCR groups in each location of the gastrointestinal tract. Previous research investigating feed conversion efficiency has revealed that 36 genera of fecal bacteria were differentially abundant between high and low FCR broilers (Singh et al. 2014).

Although it is thought that they are free from bacteria prior to birth, mammals are exposed to a variety of environments containing abundant bacteria, starting with the vagina during birth. The composition of the gut microbiota is not static and shifts over time (Palmer et al. 2007). During the weaning period, the pig fecal microbiota shifts, causing a physiologically stressful time for animals (Alain et al. 2014). After weaning, the composition of the gut microbiota continues to change until market age (Kim et al. 2011). The body weight selected in this trial was from 50 kg to near the market weight (100 kg). During this time, bacterial shifts tend to stabilize, and the diversity of bacteria between the high and low FCR groups might influence the growth of individuals. Moreover, research has shown that intestinal microbiomes and porcine growth traits are linked. Bacteria in piglet feces were clustered into two enterotype-like groups. The group dominated by *Prevotella* and *Mitsuokella* genera was significantly correlated both body weight (BW) and average daily gain (ADG) (Ramayo-Caldas et al. 2016). Therefore, analysis of intestinal microbiomes using the high and low FCR trait is reasonable.

We compared the microbial diversity of the complete intestinal tract between two groups, and found that *Lactobacillus* was prevalent in the duodenum, SMB53 was enriched in the jejunum and ileum, and *Prevotella* was dominant in all hindgut locations. The fecal microbiota had a higher proportion of the genus *Treponema* compared with other intestinal locations. *Lactobacillus* have often been considered as probiotics, while research shows that *Lactobacillus acidophilus*, *Lactobacillus fermentum,* and *Lactobacillus ingluviei* are conducive to weight gain in humans and animals. However, *Lactobacillus plantarum* and *L.Lactobacillus gasseri* tended to cause weight loss in animals (Million et al. 2012). Generally, enrichment of *Lactobacillus* is beneficial for the gastrointestinal tract. The SMB53 genus belongs to the Clostridiaceae family. Most members of this family have the ability to consume mucus- and plant-derived saccharides, such as glucose, in the gut (Wuest et al. 2011). The genus *Prevotella* contributes to degradation of mucin and plant-based carbohydrates (Lamendella et al. 2011; Pajarillo et al. 2015). A higher abundance of *Prevotella* is probably related to the presence of fructo-oligosaccharides and starch in the lower intestine (Metzler-Zebeli et al. 2013). Many species of *Treponema* were reported to be pathogenic bacteria (Stamm et al. 2009). The prevalence of the genus *Oscillospira* in the posterior intestine was probably induced by potential pathogenic bacteria, and its relative abundance might be a sign of intestinal health (Lu et al. 2016).

Many studies have focused on fecal microbes as a means of studying intestinal microbiota (Kim and Isaacson 2015), because collection is easier and does not harm the animal. The relationship of intestinal tract microbiota and fecal microbiota has been reported (Stanley et al. 2015).

Although the PCoA results showed no clear distinctions between locations, the duodenum, jejunum, and ileum were distant from other locations, regardless of the FCR group status. The Mann–Whitney test and LEfSe results suggest that certain biomarkers exist in each location of high and low FCR groups. The results from the LEfSe analyses were similar to the Mann–Whitney test (Table 2). The identified microbes were related to nutrient digestion. It is also possible that the structure of gut microbes was influenced by the variability of the FCR.

According to the bacterial abundance data, the microbes at every location were clustered in two categories (Fig. 3). Two predominant clusters were distinguished in the upper and lower intestines (including the feces). We also identified the potential functions of the gastrointestinal microbiome using PICRUSt to predict the metabolic pathways. Two predominant clusters (lower left and upper right) were also distinguished in the anterior and posterior intestine, except for the Lduodenum (Fig. 6).

Glycan and carbohydrate metabolism, transport, and catabolism functions were most abundant in the posterior intestine. The anterior intestine was enriched in metabolic pathways related to the immune response, diseases, and transcription. These indicate that the posterior intestinal microorganisms participate in the digestion and metabolism of foodstuffs. We also observed a marked difference in the bacterial third-level metabolic functions in the GIT (Gastrointestinal tract) components between groups in the different locations (Supplementary Fig. 2). Except in the duodenum, the pathway differences were analyzed by LEfSe. These different pathways could be explained by different microorganisms between high and low groups in certain positions of the intestine.

In a study of the same batch of animals (Tan et al. 2017a, b), Firmicutes and Bacteroidetes were the most abundant phyla in cecal and colonic microbiota of pigs in both groups, consistent with other studies (Kim and Isaacson 2015; Pedersen et al. 2013). The dominant genera were *Prevotella* and *Bacteroides*. By functional comparison of the high and low FCR groups in cecal and colonic microbiota, we found the microorganisms that differed in abundance were mainly related to carbohydrate metabolism. These organisms may affect the growth of the host. The cecum of individuals with high FE contained differentially abundant genes that affect the host energy absorption, mainly through the pyruvate-related metabolism pathway, such as phenylalanine metabolism, synthesis and degradation of ketone bodies, arginine and ornithine metabolism, etc. (Tan et al. 2017a, b). The different metabolic pathways were significantly enriched in the colonic microbiota of the low FE group, partly because of the larger number of genes were more abundant in the low group, and possibly due to incomplete digestion of colonic nutrients, leaving more food residues in the colon that result in greater microbial activity. The main pathway differences were related to the metabolism of cofactors and vitamins. While the colon itself does not perform digestion, microbes in the colon can digest cellulose and synthesize vitamins. The differentially expressed genes of the intestinal mucosa in the cecum and colon between the high and low FCR groups were analyzed, respectively, revealed some candidate genes that might be correlated with the FE, and the subsequent functional verification could be carried out in future (Tan et al. 2017a, b).

*Prevotella* sp. CAG:604 was the species with the most difference in both low groups of cecal and colonic microbiota. *Prevotella* sp. CAG:604 expresses proteins that are involved in nutrient and energy metabolism, such as YchF, GpmI, QueF, SpeA and Fmt, etc. The presence of fructo-oligosaccharides and starch in the lower intestine may cause a higher abundance of *Prevotella* (Metzler-Zebeli et al. 2013). There were species of *Lactobacillus* enriched in the high group of both cecal and colonic microbiota, with *Lactobacillus* spp. often considered a probiotic. Several species of *Lactobacillus* belong to the family of lactic acid bacteria (LAB), which convert carbohydrates to lactic acid by homofermentation or heterofermentation, or to acetic acid by heterofermentation (Nicholson et al. 2012). These microorganisms actively participate in the process of nutrient digestion in the lower intestine, and the nutrients are absorbed through the intestinal mucosa into the circulatory system.

More information about the microbiota and a better understanding of the complex dynamics of the gut microbial community could be used to enhance the production of livestock (Looft et al. 2012, 2014). The 16S rRNA gene sequences could be used to compare gut microbial community diversity in finishing Landrace pig intestinal digesta with high and low FCR traits. The microbiota could be manipulated better for low FCR if it is better understood, focusing on the biomarkers.

## Conclusion

Our results reveal the complex bacterial community related to FCR in porcine gastrointestinal tracts. Potential biomarkers (genera) were found in different locations of the complete intestinal tract in the high and low FCR groups, which could be of potential use to distinguish individuals for growth efficiency. Functional prediction and cluster analysis confirmed bacteria in the hindgut mainly participated in nutrient metabolism. Metabolic pathways in different locations were different between the high and low groups because of the presence of different microbes. As only four pairs of pigs were used in this study, these results need be validated using a larger cohort in the future. It will benefit pork production by facilitating the detection and alteration of the intestinal microbial community, potentially improving the growth rate of pigs.

## Electronic supplementary material

Below is the link to the electronic supplementary material.

Supplementary Table 1 Individuals selected for this study and their pedigrees. Supplementary material 1 (DOCX 14 kb)

Supplementary Fig. 1 Feed Conversion Ratio (FCR) calculated in high and low groups. Significant difference was tested of twenty individuals at the ends of the high and low FCR respectively by one way variance analysis. Supplementary material 2 (TIFF 181 kb)

Supplementary Fig. 2 Linear discriminant analysis (LDA) effect size (LEfSe) results for KEGG pathways for differentially abundant microbial features of intestinal locations. Histogram of the LDA scores computed for features differentially abundant in the jejunum (A), ileum (B), cecum (C), colon (D), rectum (E) and feces (F) among high and low groups (only genera LDA scores above 2 are shown). Supplementary material 3 (TIFF 1924 kb)

## References

[CR1] Alain BPE, Chae JP, Balolong MP, Bum KH, Kang DK (2014). Assessment of fecal bacterial diversity among healthy piglets during the weaning transition. J Gen Appl Microbiol.

[CR2] Avershina E, Frisli T, Rudi K (2013). De novo semi-alignment of 16S rRNA gene sequences for deep phylogenetic characterization of next generation sequencing data. Microbes Environ.

[CR3] Caporaso JG, Kuczynski J, Stombaugh J, Bittinger K, Bushman FD, Costello EK, Fierer N, Pena AG, Goodrich JK, Gordon JI, Huttley GA, Kelley ST, Knights D, Koenig JE, Ley RE, Lozupone CA, McDonald D, Muegge BD, Pirrung M, Reeder J, Sevinsky JR, Turnbaugh PJ, Walters WA, Widmann J, Yatsunenko T, Zaneveld J, Knight R (2010). QIIME allows analysis of high-throughput community sequencing data. Nat Methods.

[CR4] Edgar RC (2013). UPARSE: highly accurate OTU sequences from microbial amplicon reads. Nat Methods.

[CR5] Gilbert H, Bidanel JP, Billon Y, Lagant H, Guillouet P, Sellier P, Noblet J, Hermesch S (2012). Correlated responses in sow appetite, residual feed intake, body composition, and reproduction after divergent selection for residual feed intake in the growing pig. J Anim Sci.

[CR6] Jing L, Hou Y, Wu H, Miao Y, Li X, Cao J, Michael Brameld J, Parr T, Zhao S (2015). Transcriptome analysis of mRNA and miRNA in skeletal muscle indicates an important network for differential residual feed intake in pigs. Sci Rep.

[CR7] Kim HB, Isaacson RE (2015). The pig gut microbial diversity: understanding the pig gut microbial ecology through the next generation high throughput sequencing. Vet Microbiol.

[CR8] Kim HB, Borewicz K, White BA, Singer RS, Sreevatsan S, Tu ZJ, Isaacson RE (2011). Longitudinal investigation of the age-related bacterial diversity in the feces of commercial pigs. Vet Microbiol.

[CR9] Kim J, Nguyen SG, Guevarra RB, Lee I, Unno T (2015). Analysis of swine fecal microbiota at various growth stages. Arch Microbiol.

[CR10] Kozich JJ, Westcott SL, Baxter NT, Highlander SK, Schloss PD (2013). Development of a dual-index sequencing strategy and curation pipeline for analyzing amplicon sequence data on the MiSeq Illumina sequencing platform. Appl Environ Microbiol.

[CR11] Lamendella R, Domingo JW, Ghosh S, Martinson J, Oerther DB (2011). Comparative fecal metagenomics unveils unique functional capacity of the swine gut. BMC Microbiol.

[CR12] Langille MGI, Zaneveld J, Caporaso JG, McDonald D, Knights D, Reyes JA, Clemente JC, Burkepile DE, Thurber RLV, Knight R, Beiko RG, Huttenhower C (2013). Predictive functional profiling of microbial communities using 16S rRNA marker gene sequences. Nat Biotechnol.

[CR13] Looft T, Johnson TA, Allen HK, Bayles DO, Alt DP, Stedtfeld RD, Sul WJ, Stedtfeld TM, Chai B, Cole JR, Hashsham SA, Tiedje JM, Stanton TB (2012). In-feed antibiotic effects on the swine intestinal microbiome. Proc Natl Acad Sci USA.

[CR14] Looft T, Allen HK, Cantarel BL, Levine UY, Bayles DO, Alt DP, Henrissat B, Stanton TB (2014). Bacteria, phages and pigs: the effects of in-feed antibiotics on the microbiome at different gut locations. ISME J.

[CR15] Louis P, Scott KP, Duncan SH, Flint HJ (2007). Understanding the effects of diet on bacterial metabolism in the large intestine. J Appl Microbiol.

[CR16] Lozupone C, Lladser ME, Knights D, Stombaugh J, Knight R (2011). UniFrac: an effective distance metric for microbial community comparison. ISME J.

[CR17] Lu S, Zuo T, Zhang N, Shi H, Liu F, Wu J, Wang Y, Xue C, Tang Q (2016). High throughput sequencing analysis reveals amelioration of intestinal dysbiosis by squid ink polysaccharide. J Funct Foods.

[CR18] Lumpkins BS, Batal AB, Lee MD (2010). Evaluation of the bacterial community and intestinal development of different genetic lines of chickens. Poult Sci.

[CR19] Macfarlane GT, Gibson GR, Mackie RI, White BA (1997). Carbohydrate fermentation, energy transduction and gas metabolism in the human large intestine. Ecology and physiology of gastrointestinal microbes.

[CR20] Metzler-Zebeli BU, Schmitz-Esser S, Klevenhusen F, Podstatzky-Lichtenstein L, Wagner M, Zebeli Q (2013). Grain-rich diets differently alter ruminal and colonic abundance of microbial populations and lipopolysaccharide in goats. Anaerobe.

[CR21] Million M, Angelakis E, Paul M, Armougom F, Leibovici L, Raoult D (2012). Comparative meta-analysis of the effect of *Lactobacillus* species on weight gain in humans and animals. Microb Pathog.

[CR22] Nicholson JK, Holmes E, Kinross J, Burcelin R, Gibson G, Jia W, Pettersson S (2012). Host-gut microbiota metabolic interactions. Science.

[CR23] Pajarillo EAB, Chae JP, Balolong MP, Kim HB, Seo K, Kang D (2015). Characterization of the fecal microbial communities of duroc pigs using 16S rRNA gene pyrosequencing. Asian Aust J Anim.

[CR24] Palmer C, Bik EM, DiGiulio DB, Relman DA, Brown PO (2007). Development of the human infant intestinal microbiota. PLoS Biol.

[CR25] Parks BW, Nam E, Org E, Kostem E, Norheim F, Hui ST, Pan C, Civelek M, Rau CD, Bennett BJ, Mehrabian M, Ursell LK, He A, Castellani LW, Zinker B, Kirby M, Drake TA, Drevon CA, Knight R, Gargalovic P, Kirchgessner T, Eskin E, Lusis AJ (2013). Genetic control of obesity and gut microbiota composition in response to high-fat, high-sucrose diet in mice. Cell Metab.

[CR26] Pedersen R, Ingerslev H, Sturek M, Alloosh M, Cirera S, Christoffersen BO, Moesgaard SG, Larsen N, Boye M (2013). Characterisation of gut microbiota in ossabaw and gottingen minipigs as models of obesity and metabolic syndrome. PLoS ONE.

[CR27] Ramayo-Caldas Y, Mach N, Lepage P, Levenez F, Denis C, Lemonnier G, Leplat J, Billon Y, Berri M, Dore J, Rogel-Gaillard C, Estelle J (2016). Phylogenetic network analysis applied to pig gut microbiota identifies an ecosystem structure linked with growth traits. ISME J.

[CR28] Schwiertz A, Taras D, Schaefer K, Beijer S, Bos NA, Donus C, Hardt PD (2010). Microbiota and SCFA in lean and overweight healthy subjects. Obesity.

[CR29] Segata N, Izard J, Waldron L, Gevers D, Miropolsky L, Garrett WS, Huttenhower C (2011). Metagenomic biomarker discovery and explanation. Genome Biol.

[CR30] Singh KM, Shah T, Deshpande S, Jakhesara SJ, Koringa PG, Rank DN, Joshi CG (2012). High through put 16S rRNA gene-based pyrosequencing analysis of the fecal microbiota of high FCR and low FCR broiler growers. Mol Biol Rep.

[CR31] Singh KM, Shah TM, Reddy B, Deshpande S, Rank DN, Joshi CG (2014). Taxonomic and gene-centric metagenomics of the fecal microbiome of low and high feed conversion ratio (FCR) broilers. J Appl Genet.

[CR32] Stamm LV, Walker RL, Read DH (2009). Genetic diversity of bovine ulcerative mammary dermatitis-associated treponema. Vet Microbiol.

[CR33] Stanley D, Geier MS, Chen H, Hughes RJ, Moore RJ (2015). Comparison of fecal and cecal microbiotas reveals qualitative similarities but quantitative differences. BMC Microbiol.

[CR34] Tan Z, Wang Y, Yang T, Xing K, Ao H, Chen S, Zhang F, Zhao X, Liu J, Wang C (2017). Differentially expressed genes in the caecal and colonic mucosa of Landrace finishing pigs with high and low food conversion ratios. Sci Rep.

[CR35] Tan Z, Yang T, Wang Y, Xing K, Zhang F, Zhao X, Ao H, Chen S, Liu J, Wang C (2017). Metagenomic analysis of cecal microbiome identified microbiota and functional capacities associated with feed efficiency in landrace finishing pigs. Front Microbiol.

[CR36] Turnbaugh PJ, Ley RE, Mahowald MA, Magrini V, Mardis ER, Gordon JI (2006). An obesity-associated gut microbiome with increased capacity for energy harvest. Nature.

[CR37] Wuest PK, Horn MA, Drake HL (2011). Clostridiaceae and enterobacteriaceae as active fermenters in earthworm gut content. ISME J.

[CR38] Yan W, Sun C, Yuan J, Yang N (2017). Gut metagenomic analysis reveals prominent roles of Lactobacillus and cecal microbiota in chicken feed efficiency. Sci Rep.

[CR39] Yang L, Bian G, Su Y, Zhu W (2014). Comparison of faecal microbial community of Lantang, Bama, Erhualian, Meishan, Xiaomeishan, Duroc, Landrace, and Yorkshire Sows. Asian Aust J Anim.

[CR40] Yang H, Huang X, Fang S, Xin W, Huang L, Chen C (2016). Uncovering the composition of microbial community structure and metagenomics among three gut locations in pigs with distinct fatness. Sci Rep.

