# Supplementary material for: Differences in gut microbiota composition in finishing Landrace pigs with low and high feed conversion ratios
Source: Antonie Van Leeuwenhoek. 2018 Mar 1;111(9):1673–85. doi: 10.1007/s10482-018-1057-1 (PMC6097733; doi:10.1007/s10482-018-1057-1)
Supplement: Supplementary file 1 — Individuals selected for this study and their pedigrees. Supplementary material 1 (DOCX 14 kb) [file 10482_2018_1057_MOESM1_ESM.docx]

**Supplementary Table 1 Individuals selected for this study and their pedigree**

| Group | ID | Status | Sample | Initial BW (Kg) | Final BW (kg) | FCR | Father ID | Mother ID |
| --- | --- | --- | --- | --- | --- | --- | --- | --- |
| Lgroup | 124110 | Half-sib | L1 | 50.6 | 81.8 | 3.12 | LLDA27813008791 | LLNHPF714105513 |
| Hgroup | 143014 |  | H1 | 49.4 | 92.5 | 2.02 | LLDA27813008791 | LLNHPF713043113 |
| Lgroup | 144013 | Half-sib | L2 | 50.1 | 86.2 | 2.8 | LLNHPF711011101 | LLNHPF713012411 |
| Hgroup | 143106 |  | H2 | 50.5 | 93.9 | 1.99 | LLNHPF711011101 | LLNHPF712131314 |
| Lgroup | 126614 | Full-sib | L3 | 51.5 | 86.8 | 2.67 | LLDA27813008791 | LLNHPF712304013 |
| Hgroup | 126606 |  | H3 | 50.2 | 92.5 | 2.03 | LLDA27813008791 | LLNHPF712304013 |
| Lgroup | 130506 | Full-sib | L4 | 49.7 | 84.3 | 2.79 | LLNHPF712246400 | LLNHPF714116103 |
| Hgroup | 130504 |  | H4 | 51.5 | 92 | 2.09 | LLNHPF712246400 | LLNHPF714116103 |
