# Supplementary figures and images for: Differences in gut microbiota composition in finishing Landrace pigs with low and high feed conversion ratios
Source: Antonie Van Leeuwenhoek. 2018 Mar 1;111(9):1673–85. doi: 10.1007/s10482-018-1057-1 (PMC6097733; doi:10.1007/s10482-018-1057-1)

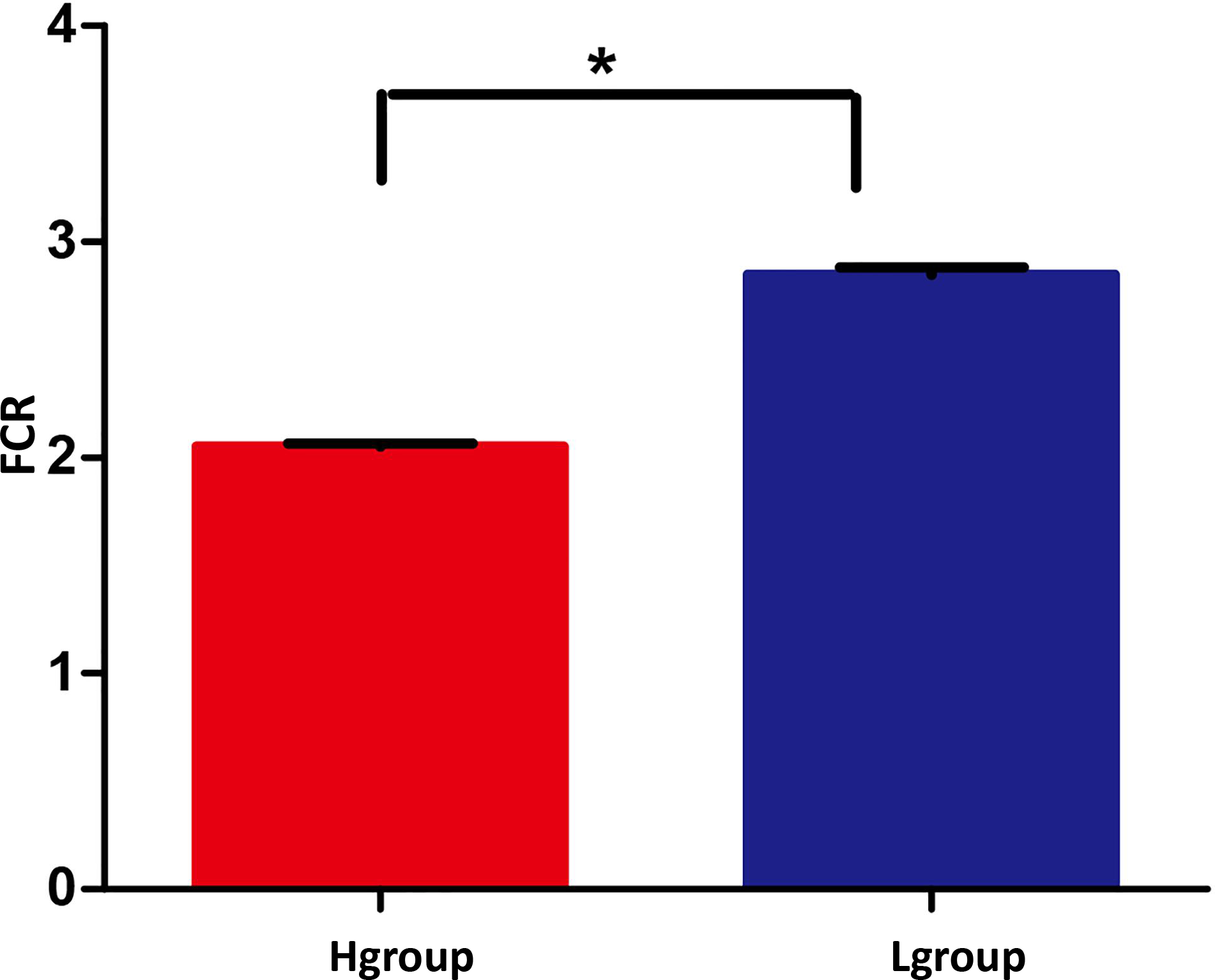

Supplement: Supplementary file 2 — Feed Conversion Ratio (FCR) calculated in high and low groups. Significant difference was tested of twenty individuals at the ends of the high and low FCR respectively by one way variance analysis. Supplementary material 2 (TIFF 181 kb) [file 10482_2018_1057_MOESM2_ESM.tif]

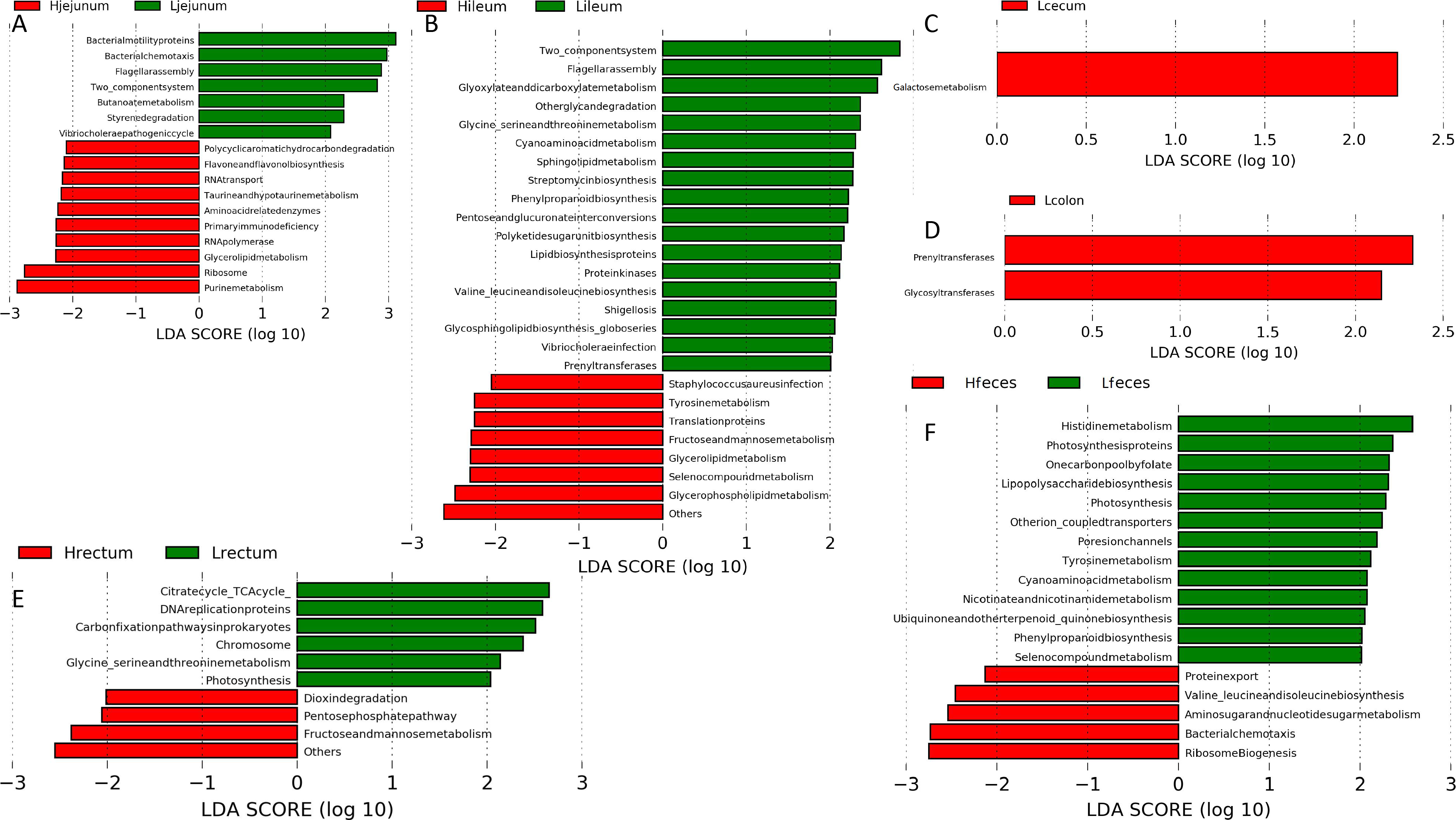

Supplement: Supplementary file 3 — Linear discriminant analysis (LDA) effect size (LEfSe) results for KEGG pathways for differentially abundant microbial features of intestinal locations. Histogram of the LDA scores computed for features differentially abundant in the jejunum (A), ileum (B), cecum (C), colon (D), rectum (E) and feces (F) among high and low groups (only genera LDA scores above 2 are shown). Supplementary material 3 (TIFF 1924 kb) [file 10482_2018_1057_MOESM3_ESM.tif]
